# Immediate Outcomes of Aortic Valve Neocuspidization with Glutaraldehyde-treated Autologous Pericardium: a Multicenter Study

**DOI:** 10.21470/1678-9741-2020-0019

**Published:** 2020

**Authors:** Vagram Arutyunyan, Igor Chernov, Roman Komarov, Yuriy Sinelnikov, Bakytbek Kadyraliev, Soslan Enginoev, Maxim Tcheglov, Alisher Ismailbaev, Aleksey Baranov, Fatali Ashurov, Marie-Annick Clavel, Philippe Pibarot, Michel Pompeu B. O. Sá, Alexander Weymann, Konstantin Zhigalov

**Affiliations:** 1 Department of Cardiovascular Surgery, S. G. Sukhanov Federal Center of Cardiovascular Surgery, E. A. Vagner Perm State Medical University, Perm, Russia.; 2 Department of Cardiac Surgery, Federal Center for Cardiovascular Surgery, Astrakhan State Medical University, Astrakhan, Russia.; 3 Department of Cardiovascular Surgery, I. M. Sechenov University Hospital, First Moscow State Medical University, Moscow, Russia.; 4 Department of Cardiac Surgery, University Hospital of Bashkir State Medical University, Ufa, Russia.; 5 Institut Universitaire de Cardiologie et de Pneumologie de Québec, Quebec Heart and Lung Institute, Quebec, Canada.; 6 Division of Cardiovascular Surgery, Pronto-Socorro Cardiológico de Pernambuco - PROCAPE, University of Pernambuco - UPE, Recife, Brazil.; 7 Department of Thoracic and Cardiovascular Surgery, West German Heart and Vascular Center Essen, University Hospital of Essen, University Duisburg-Essen, Essen, Germany.

**Keywords:** Aortic Valve, Glutaral, Hospital Mortality, Feasibility Studies, Aortic Valve, Stenosis, Endocarditis, Bacterial, Echocardiography, Pericardium

## Abstract

**Objective:**

To determine the feasibility of aortic valve neocuspidization (AVNeo) with glutaraldehyde-treated autologous pericardium.

**Methods:**

One hundred and seventy (170) AVNeo (84 males/86 females) were performed from January 2017 through March 2019 in three centers. All the records were prospectively collected and retrospectively reviewed.

**Results:**

Most of the patients were older than 60 years and over 95% were operated for aortic stenosis. Preoperatively, pressure gradients were 69.9±21.3 mmHg for patients with aortic stenosis, and the surgical annular diameter was 21.0±2.0 mm for all patients. Effective orifice area (EOA) and indexed EOA (iEOA) averaged 0.7±0.3 cm^2^ and 0.4±0.2 cm^2^/m^2^ for patients with aortic stenosis before surgery, respectively. There was no conversion to prosthetic aortic valve replacement. Eight patients needed reoperation for bleeding, but no patient needed reoperation due to early infective endocarditis. There were five in-hospital deaths due to noncardiac cause. Compared to preoperative echocardiographic measurements, postoperative peak pressure gradient decreased significantly (-58.7±1.7 mmHg; *P*<0.001) and reached 11.2±5.6 mmHg, and mean pressure gradient also decreased significantly (-36.8±1.1 mmHg; *P*<0.001) and reached 6.0±3.5 mmHg. Accordingly, EOA and iEOA increased significantly 2.0 cm^2^ and 1.0 cm^2^/m^2^ (both *P*<0.001) to reach 2.7±0.6 cm^2^ and 1.4±0.3 cm^2^/m^2^ after surgery, respectively, with minimal significant aortic regurgitation (0.6% > mild).

**Conclusion:**

AVNeo is feasible and reproducible with good clinical results. Hemodynamically, AVNeo produces immediate postoperative low-pressure gradients, large EOA, and minimal regurgitation of the aortic valve. Further studies are necessary to evaluate mid- and long-term evolution.

**Table:** 

Abbreviations, acronyms & symbols				
AR	= Aortic regurgitation		iEOA	= Indexed effective orifice area
AS	= Aortic stenosis		LAA	= Left atrial appendage
AVNeo	= Aortic valve neocuspidization		LVEF	= Left ventricular ejection fraction
AVR	= Aortic valve replacement		NYHA	= New York Heart Association
COPD	= Chronic obstructive pulmonary disease		PG	= Pressure gradients
EOA	= Effective orifice area		PHV	= Prosthetic heart valves
EuroSCORE	= European System for Cardiac Operative Risk Evaluation		PPM	= Prosthesis-patient mismatch
ICU	= Intensive care unit		SD	= Standard deviation

## INTRODUCTION

Aortic valve replacement (AVR) is the gold standard treatment for aortic valve diseases. However, most of prosthetic valves have a stent structure for suturing and fixing to the aortic valve annulus, which culminates in less mobility and decrease of the annulus size and increase in the postoperative pressure gradients. Furthermore, there are also cases that result in prosthesis-patient mismatch (PPM) both in surgical AVR^[1,2]^ and in transcatheter aortic valve implantation^[3]^. Additionally, in patients with a small aortic annulus, AVR may be difficult and aortic root enlargement is necessary to implant the largest possible prosthesis in order to avoid postoperative PPM^[4]^.

It has been over 10 years since Ozaki et al.^[5]^ started to perform the aortic valve neocuspidization (AVNeo) procedure, which is a surgical procedure consisting of the complete resection of the aortic cusps and the direct suture of the glutaraldehyde-treated autologous pericardium to the aortic annulus. This technique has been applied to various aortic valve pathologies with good mid-term results^[5-9]^.

In this study, we aimed to elucidate whether AVNeo is an acceptable and reproducible option by evaluating the immediate postoperative clinical and echocardiographic outcomes.

## METHODS

### Study Design

**Fig. 1A:**
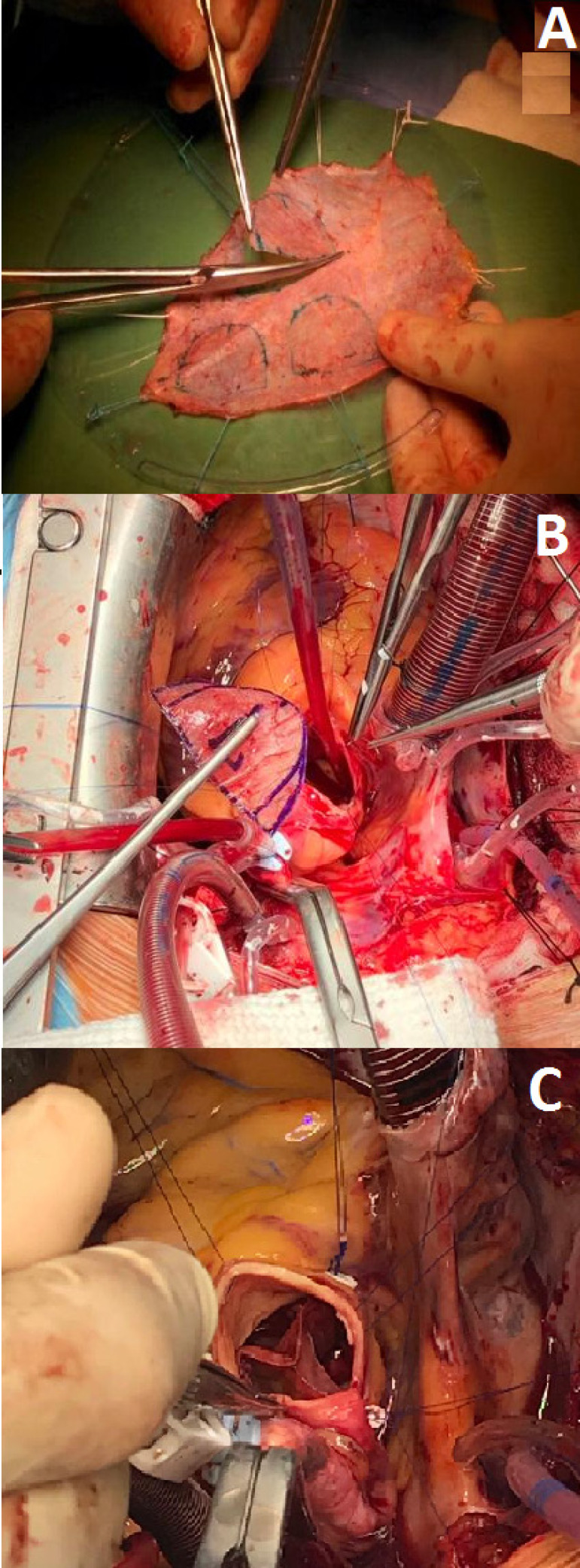
Trimming of treated autologous pericardium. Fig. 1B – Suture of the neocusp to the aortic annulus. Fig. 1C – Aortic valve neocuspidization, a final view.

We carried out a prospective multicenter study in men and women with aortic valve diseases selected for AVNeo procedure. The study was approved by the local ethics committee of each participating institution. All patients who were willing to undergo AVNeo were evaluated for in-hospital clinical results. Detailed descriptions of the surgical techniques for AVNeo have been previously published^[5-11]^. Figures 1A, 1B, and 1C depict the main steps of the procedure.

### Echocardiographic Measurement

All patients underwent an echocardiographic evaluation of the aortic valve before and after the procedure. Peak and mean gradient were calculated by the modified Bernouilli equation. Effective orifice area (EOA) (cm^2^) was calculated with the continuity equation, with the use of stroke volume estimated by the left ventricular outflow tract diameter and velocity time integral within the left ventricular outflow tract. EOA was indexed (iEOA) by the body surface area (m^2^). Aortic regurgitation (AR) was graded using an integrative approach including structural, Doppler, quantitative, and qualitative parameters as recommended by the American Society of Echocardiography guidelines^[12]^.

### Endpoints

The primary endpoints were operative mortality and hemodynamic performance of AVNeo through echocardiographic evaluation, which was done during the hospital stay prior to discharge. Secondary endpoints were perioperative adverse events. All the patients were followed during their hospital stay.

### Statistical Analysis

Continuous data are presented as means ± standard deviation (SD) after confirming normal distribution with Shapiro-Wilk test. Categorical variables are expressed as numbers and percentages. Between-group differences were evaluated using the independent Student’s *t*-test for normally distributed data and the Mann-Whitney U test for non-normally distributed data. Post *vs*. preprocedure variation was tested with Levene’s test and F-test. All analyses were performed using the IBM SPSS Statistics software (version 22.0, SPSS Inc., Chicago, Illinois, United States of America). *P*-values < 0.05 were considered to indicate a statistically significant difference.

## RESULTS

### Population

Among the patients, as shown in Table 1, there were 84 males and 86 females. Mean age was 64 (range, 23-86) years. Regarding the age distribution, most of the patients were older than 60 years.

**Table 1:** Patients' baseline characteristics and risk factors.

Characteristics		Total
Demographic data	Patients, n (%)	170 (100%)
	Age, years (mean±SD)	64.1±9.7
	Females/males, %	50.6/49.4
	Body mass index, kg/m^2^(mean±SD)	29.5±5.5
	Body surface area, m^2^(mean±SD)	1.9±0.2
	NYHA class (mean±SD)	2.7±0.6
	EuroSCORE II, % (median - range)	2.6 (1.7 - 5.1)
Comorbidities, n (%)	Coronary artery disease	61 (35.9%)
	Diabetes	26 (15.3%)
	Renal dysfunction	4 (2.4%)
	COPD	22 (12.9%)
	Peripheral vascular disease	11 (6.5%)
	Previous sternotomy	6 (3.5%)
Surgery indication, n (%)	Severe aortic stenosis	162 (95.3%)
	Severe aortic regurgitation	3 (1.8%)
	Infective endocarditis (no AS, no AR)	5 (2.9%)
Echocardiographic data	LVEF, % (mean±SD)	58.3±9.4
	Average peak pressure gradient (mmHg; ±SD)	69.9±21.3
	Average mean pressure gradient (mmHg; ±SD)	42.8±13.4
	Average annulus diameter (mm; ±SD)	21.0±2.0
	Aortic annulus with diameter < 23 mm, n (%)	134 (78.8%)
	Aortic annulus with diameter ≤ 21 mm, n (%)	106 (62.3%)
	Aortic annulus with diameter ≤ 19 mm, n (%)	40 (23.5%)
Morphology of the aortic valve, n (%)	Tricuspid	118 (69.9%)
	Bicuspid	52 (30.1%)

AR=aortic regurgitation; AS=aortic stenosis; COPD=chronic obstructive pulmonary disease; EuroSCORE=European System for Cardiac Operative Risk Evaluation; LVEF=left ventricular ejection fraction; NYHA=New York Heart Association; SD=standard deviation

One hundred sixty-two (95%) patients had aortic stenosis (AS), three patients had AR, and five had early infective endocarditis. Fifty-two (31%) patients had bicuspid aortic valve. Preoperative echocardiography showed an average peak pressure gradient through the aortic valve of 69.9±21.3 mmHg for patients with AS and a surgical annular diameter of 21.0±2.0 mm for all patients. EOA and iEOA averaged 0.7±0.3 cm^2^ and 0.4±0.2 cm^2^/m^2^ before surgery, respectively.

### Perioperative Clinical Events

A total of 94 (55%) patients had isolated AVNeo during the study period and the other 76 (45%) patients underwent concomitant procedures (Table 2). There was no conversion to classical prosthetic AVR. Mean cardiopulmonary bypass time was 112.2±38.9 min and aortic cross-clamp time was 86.2±22.7 min (Table 3). Eight patients needed reoperation for bleeding and two pacemaker implantations, but no patient needed reoperation due to early infective endocarditis. No thromboembolic events were recorded. There were five in-hospital deaths due to noncardiac cause.

**Table 2:** Operative data.

Characteristics	Total
Durations, min (mean ± SD)	
Procedure time	221.3±45.2
Cardiopulmonary bypass	112.2±38.9
Aortic cross-clamp	86.2±22.7
Conventional sternotomy, n (%)	164 (96.5%)
Minimally invasive approach, n (%)	6 (3.5%)
Upper T-shaped mini-sternotomy	5 (2.9%)
Upper J-shaped mini-sternotomy	1 (0.6%)
Isolated aortic valve reconstruction, n (%)	94 (55.3%)
Concomitant procedures, n (%)	76 (44.7%)
Coronary artery bypass graft	44 (25.9%)
Replacement of ascending aorta	17 (10%)
Aortic root surgery	10 (5.9%)
Mitral valve surgery	6 (3.5%)
Tricuspid valve surgery	5 (2.9%)
Atrium ablation with LAA closure	4 (2.4%)
Carotid endarterectomy	3 (1.8%)

LAA=left atrial appendage; SD=standard deviation

**Table 3:** Postoperative events.

Characteristics		Total
Operative mortality, %		5 (2.9%)
Reoperation for bleeding, n (%)		8 (4.7%)
Aortic regurgitation	None	131 (77.1%)
	Mild	38 (22.3%)
	Moderate	1 (0.6%)
	Severe	0 (0%)
Permanent pacemaker implantation, n (%)		2 (1.2%)
Disabling stroke, n (%)		0 (0%)
Endocarditis, n (%)		0 (0%)
Sternal wound infection, n (%)		9 (5.3%)
Sepsis, n (%)		1 (0.9%)
Acute renal failure, n (%)		4 (2.4%)
Ventilation on ICU, hours (median - range)		12 (9 - 15)
ICU stay, days (median - range)		1 (1 - 1)
Hospital stay, days (median - range)		13 (10 - 16)

ICU=intensive care unit

### Postoperative Echocardiography

**Fig. 2A:**
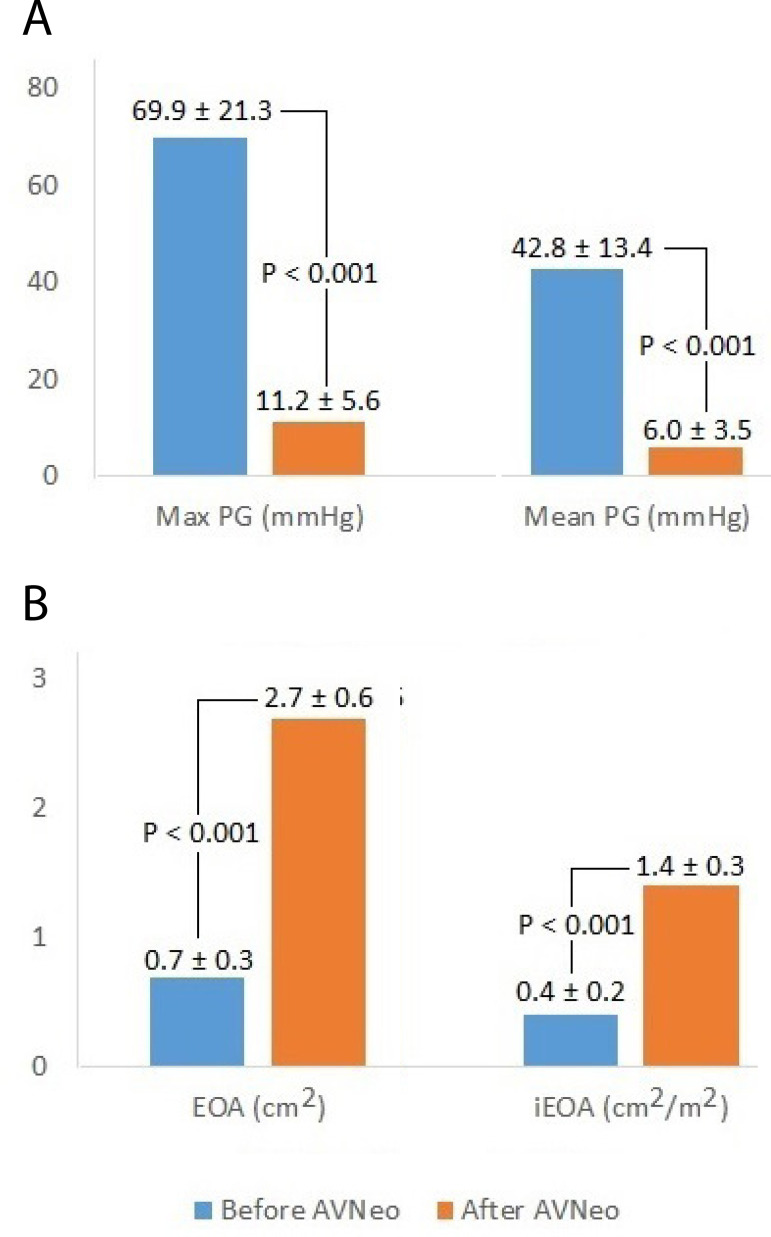
Comparison between pre- and postoperative echocardiographic data regarding maximum and mean pressure gradients (PG) ± standard deviation. AVNeo=aortic valve neocuspidization. **Fig. 2B** – Comparison between pre- and postoperative echocardiographic data regarding effective orifice area (EOA) and indexed effective orifice area (iEOA). AVNeo=aortic valve neocuspidization

Average peak and mean pressure gradients were 11.2±5.6 mmHg and 6.0±3.5 mmHg (mean±SD), respectively, after surgery. Thus, both decreased significantly (difference in means±standard error; peak gradient: -58.7±1.7 mmHg, *P*<0.001; and mean gradient: -36.8±1.1 mmHg, *P*<0.001) in comparison to preoperative measures (Figure 2A).

**Fig. 3:**
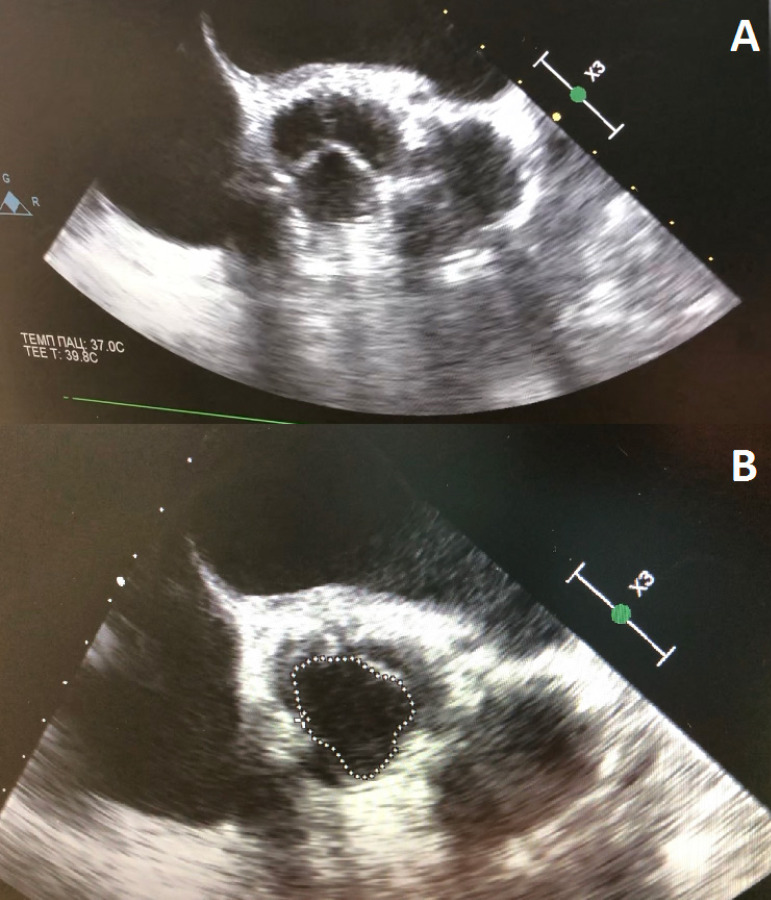
Postoperative echocardiographic views in diastole (A) and systole (B).

EOA and iEOA averaged 2.7±0.6 cm^2^ and 1.4±0.3 cm^2^/m^2^ after surgery, respectively. They significantly increased 2.0±0.1 cm^2^ and 1.0±0.1 cm^2^/m^2^, respectively, in comparison to preoperative measures (Figure 2B). Interestingly, we observed no PPM after the procedure (*i.e*., all iEOA > 0.85 cm^2^/m^2^). After AVNeo procedure, 38 (22.3%) patients had a mild AR and one (0.6%) had moderate AR. The postoperative echocardiographic views are presented in Figures 3A and 3B.

## DISCUSSION

The main finding of our study was that AVNeo is a feasible procedure with: 1) good perioperative outcome (2.9% operative death observed in a population with 2.6% expected operative death calculated by the European System for Cardiac Operative Risk Evaluation - EuroSCORE II), 2) low perioperative complications, and 3) excellent postoperative hemodynamic results (mean gradient post-AVNeo 6 mmHg, only 0.6% moderate AR).

Whereas AVR with prosthetic heart valves (PHV) has been considered the best option for treatment of aortic valve diseases, many unanswered questions surround intraoperative sizing and labeling of these prostheses, making optimal intraoperative selection challenging. According to a document^[13]^ from the European Association for Cardio-Thoracic Surgery - EACTS, the Society of Thoracic Surgeons - STS, and the American Association for Thoracic Surgery - AATS Valve Labelling Task Force, these questions include: non-uniform or incomplete reporting of PHV materials and physical dimensions in the instructions for use; unclear definition of labeled valve size and inconsistencies between size dimensions and labeled valve size; non-uniform marking of PHV support structures; lack of robust information on *in vivo* hemodynamic performance and no information available regarding hemodynamic performance on package labels; lack of uniform tools backed by solid evidence to prevent PPM; and lack of good-quality, robust clinical data on PHV thrombogenicity.

This situation has received many calls for action, but no solution has been achieved so far. In this scenario, AVNeo arises as an attractive option owing to its low cost, universal indications without any limit in annulus size nor any need for anticoagulation, and its potentially excellent hemodynamics.

The most important findings of our study were, in addition to low operative mortality, the statistically significant decreases in pressure gradients and increase in EOA and iEOA after AVNeo. We would like to highlight the fact that these findings were consistent regardless of the annulus size. We also found no patient in the postoperative period with values of iEOA < 0.85cm^2^/m^2^, which means no postoperative PPM. These observations might underlie the maintenance of the characteristics of the aortic annulus due to the absence of stent structures.

Iida et al.^[14]^ performed AVNeo for various aortic valve pathologies in 147 patients from December 2010 to October 2017. Of these patients, the aortic annulus dimensions were measured in 25 patients who underwent AVNeo for aortic valve disease as follow-up examination and compared with those measured in 15 patients who had normal aortic valves. No significant differences in the aortic annulus dimensions were observed between the patients who had undergone AVNeo and those who had normal aortic valves. The authors concluded that the movement of the aortic annulus after AVNeo is comparable with that of the aortic annulus of a normal aortic valve; thus, AVNeo can be regarded as a more physiological operation in that it maintains the characteristics of the aortic valve similar to those of a normal aortic valve (which in turn does not happen when patients undergo AVR).

Similarly, Yamamoto et al.^[15]^ measured aortic annular dimensions using electrocardiography-gated multidetector computed tomography in 23 patients. The sample included eight patients who had undergone AVNeo, 10 patients with normal aortic valves, and five patients who had undergone AVR. Postoperative peak pressure gradients for the AVNeo and AVR groups were compared. No statistically significant differences in annulus variation were observed between patients who had undergone AVNeo and those with normal aortic valves. Annular area was larger during systole than during diastole in both groups. Postoperative peak pressure gradients were significantly lower in the AVNeo group than in the AVR group. The results of this study demonstrated that aortic annular dimensions after AVNeo are similar to the dimensions of normal aortic valves. Lower postoperative pressure gradients might underlie the observed differences.

In another study, Iida et al.^[16]^ performed AVNeo for AS in 57 patients from December 2010 to June 2017. Their mean age was 77.5±8.8 years. Preoperative echocardiography revealed an average peak pressure gradient of 89±32.9 mmHg that decreased to 22±10.7 mmHg one week after the procedure and 19.2±9.7 mmHg 20 months after the procedure. There were no conversions to AVR. There were two noncardiac-related deaths. Two patients underwent reoperation owing to infective endocarditis and recurrent AR. The mean follow-up period was 30.4±20.8 months. The freedom from reoperation rates was 98.1 and 95.3% at 12 and 81 months of follow-up, respectively.

Mourad et al.^[17]^ carried out a prospective single-center study including 52 consecutive patients who underwent AVNeo between September 2015 and March 2017 using autologous pericardium in 16 patients. Most patients presented with AS or endocarditis. The mean age was 60±14 years. Early outcomes included one stroke, two patients needing short-term dialysis, and one death. During follow-up (mean 11.2±4.8 months), trace AR was observed in four patients; the mean pressure gradient was 6.8±2.9 mmHg. Three patients died later (of noncardiac reasons) and five patients needed reoperation due to endocarditis.

Krane et al.^[18]^ operated on 77 patients undergoing AVNeo following the Ozaki procedure between October 2016 and August 2018. Their mean age was 54.9±16.5 years; AS was present in 84.4% and insufficiency in 15.6% of the patients. At 1.76-year follow-up, freedom from reoperation was 97.4%. Two patients (2.6%) presented with moderate to severe aortic insufficiency after the procedure. Both received a prosthetic AVR within the same hospital stay. At discharge, mean pressure gradient was 9.3±4.2 mmHg, which decreased to a mean aortic gradient of 1.6±3.4 mmHg at six to 12 months. The authors concluded that AVNeo following the Ozaki procedure revealed excellent early hemodynamic results in terms of EOA, pressure gradients, and PPM. As we can see, surgeons from all over the world have achieved good initial results by reproducing this surgical procedure in their own populations, which makes AVNeo seem even more promising.

### Study Limitations

The major limitation of the current study is the lack of mid- and long-term follow-ups. We will continue to monitor the results for a longer follow-up period with a larger number of patients.

## CONCLUSION

The AVNeo procedure is feasible and reproducible and it has shown good immediate results. Our findings show that aortic valve tricuspidization with glutaraldehyde-treated autologous pericardium produces immediate postoperative low-pressure gradients, large EOA, and minimal regurgitation of the aortic valve. Further studies are required to assess mid- and long-term evolution of the neo-aortic valve.

**Table:** 

Authors' roles & responsibilities	
VA	Substantial contributions to the conception or design of the work; or the acquisition, analysis, or interpretation of data for the work; drafting the work or revising it critically for important intellectual content; agreement to be accountable for all aspects of the work in ensuring that questions related to the accuracy or integrity of any part of the work are appropriately investigated and resolved; final approval of the version to be published
IC	Substantial contributions to the conception or design of the work; or the acquisition, analysis, or interpretation of data for the work; drafting the work or revising it critically for important intellectual content; agreement to be accountable for all aspects of the work in ensuring that questions related to the accuracy or integrity of any part of the work are appropriately investigated and resolved; final approval of the version to be published
RK	Substantial contributions to the conception or design of the work; or the acquisition, analysis, or interpretation of data for the work; drafting the work or revising it critically for important intellectual content; agreement to be accountable for all aspects of the work in ensuring that questions related to the accuracy or integrity of any part of the work are appropriately investigated and resolved; final approval of the version to be published
YS	Substantial contributions to the conception or design of the work; or the acquisition, analysis, or interpretation of data for the work; drafting the work or revising it critically for important intellectual content; agreement to be accountable for all aspects of the work in ensuring that questions related to the accuracy or integrity of any part of the work are appropriately investigated and resolved; final approval of the version to be published
BK	Substantial contributions to the conception or design of the work; or the acquisition, analysis, or interpretation of data for the work; drafting the work or revising it critically for important intellectual content; agreement to be accountable for all aspects of the work in ensuring that questions related to the accuracy or integrity of any part of the work are appropriately investigated and resolved; final approval of the version to be published
SE	Substantial contributions to the conception or design of the work; or the acquisition, analysis, or interpretation of data for the work; drafting the work or revising it critically for important intellectual content; agreement to be accountable for all aspects of the work in ensuring that questions related to the accuracy or integrity of any part of the work are appropriately investigated and resolved; final approval of the version to be published
MT	Substantial contributions to the conception or design of the work; or the acquisition, analysis, or interpretation of data for the work; drafting the work or revising it critically for important intellectual content; agreement to be accountable for all aspects of the work in ensuring that questions related to the accuracy or integrity of any part of the work are appropriately investigated and resolved; final approval of the version to be published
AI	Substantial contributions to the conception or design of the work; or the acquisition, analysis, or interpretation of data for the work; drafting the work or revising it critically for important intellectual content; agreement to be accountable for all aspects of the work in ensuring that questions related to the accuracy or integrity of any part of the work are appropriately investigated and resolved; final approval of the version to be published
AB	Substantial contributions to the conception or design of the work; or the acquisition, analysis, or interpretation of data for the work; drafting the work or revising it critically for important intellectual content; agreement to be accountable for all aspects of the work in ensuring that questions related to the accuracy or integrity of any part of the work are appropriately investigated and resolved; final approval of the version to be published
FA	Substantial contributions to the conception or design of the work; or the acquisition, analysis, or interpretation of data for the work; drafting the work or revising it critically for important intellectual content; agreement to be accountable for all aspects of the work in ensuring that questions related to the accuracy or integrity of any part of the work are appropriately investigated and resolved; final approval of the version to be published
MAC	Substantial contributions to the conception or design of the work; or the acquisition, analysis, or interpretation of data for the work; drafting the work or revising it critically for important intellectual content; agreement to be accountable for all aspects of the work in ensuring that questions related to the accuracy or integrity of any part of the work are appropriately investigated and resolved; final approval of the version to be published
PP	Substantial contributions to the conception or design of the work; or the acquisition, analysis, or interpretation of data for the work; drafting the work or revising it critically for important intellectual content; agreement to be accountable for all aspects of the work in ensuring that questions related to the accuracy or integrity of any part of the work are appropriately investigated and resolved; final approval of the version to be published
MPBOS	Substantial contributions to the conception or design of the work; or the acquisition, analysis, or interpretation of data for the work; drafting the work or revising it critically for important intellectual content; agreement to be accountable for all aspects of the work in ensuring that questions related to the accuracy or integrity of any part of the work are appropriately investigated and resolved; final approval of the version to be published
AW	Substantial contributions to the conception or design of the work; or the acquisition, analysis, or interpretation of data for the work; drafting the work or revising it critically for important intellectual content; agreement to be accountable for all aspects of the work in ensuring that questions related to the accuracy or integrity of any part of the work are appropriately investigated and resolved; final approval of the version to be published
KZ	Substantial contributions to the conception or design of the work; or the acquisition, analysis, or interpretation of data for the work; drafting the work or revising it critically for important intellectual content; agreement to be accountable for all aspects of the work in ensuring that questions related to the accuracy or integrity of any part of the work are appropriately investigated and resolved; final approval of the version to be published
